# Association between metabolic syndrome and stroke: a population based cohort study

**DOI:** 10.1186/s12902-023-01383-6

**Published:** 2023-06-06

**Authors:** Amir Moghadam-Ahmadi, Narjes Soltani, Fatemeh Ayoobi, Zahra Jamali, Tabandeh Sadeghi, Nazanin Jalali, Alireza Vakilian, Mohammad Amin Lotfi, Parvin khalili

**Affiliations:** 1 Non-Communicable Diseases Research Center, Rafsanjan University of Medical Sciences, Rafsanjan, Iran; 2 Neuro-immunology Research Scholar, Jefferson Hospital for Neuroscience, Thomas Jefferson University, Philadelphia, PA US; 3 Neurology Department, School of Medicine, Rafsanjan University of Medical Sciences, Rafsanjan, Iran; 4 Physiology-Pharmacology Research Center, Rafsanjan University of Medical Science, Rafsanjan, Iran; 5 Occupational Safety and Health Research Center, NICICO, World Safety Organization and Rafsanjan University of Medical Sciences, Rafsanjan, Iran; 6 Pistachio Safety Research Center, Rafsanjan University of Medical Sciences, Rafsanjan, Iran; 7 Clinical Research Development Unit (CRDU), Niknafs Hospital, Rafsanjan University of Medical Sciences, Rafsanjan, Iran; 8 Department of Pediatric Nursing, School of Nursing and Midwifery, Rafsanjan University of Medical Sciences, Rafsanjan, Iran; 9 Clinical Research Development Unit, Ali-Ibn Abi-Talib Hospital (CRDU), Rafsanjan University of Medical Sciences, Rafsanjan, Iran; 10 Department of internal Medicine, Ali-Ibn Abi-Talib Hospital, Rafsanjan University of Medical Sciences, Rafsanjan, Iran; 11 Department of Epidemiology, School of Public Health, Social Determinants of Health Research Center, Rafsanjan University of Medical Sciences, Rafsanjan, Iran

**Keywords:** Metabolic syndrome, Stroke, Rafsanjan Cohort Study (RCS), Prospective epidemiological research studies in Iran (PERSIAN cohort study)

## Abstract

Both metabolic syndrome (MetS) and stroke are associated with increased risk of mortality. Here, we aimed to assess the prevalence of MetS among adults using three definitions (Adult Treatment Panel III (ATP-III), International Diabetes Federation (IDF) and IDF ethnic specific cut-off for Iranian criteria) and its association with stroke. We performed a cross-sectional study of a total of 9991 adult participants of Rafsanjan Cohort Study (RCS), as part of the Prospective epidemiological research studies in Iran (PERSIAN cohort study). The MetS prevalence was evaluated in participants according to the different criteria. Multivariate logistic regression analyses were conducted to assess the association between three definitions of MetS with stroke. We found that MetS was significantly associated with higher odds of stroke according to NCEP-ATP III (odds ratio (OR): 1.89, 95% confidence interval (CI) 1.30–2.74), international IDF (OR:1.66, 95% CI: 1.15–2.40) and Iranian IDF (OR:1.48, 95% CI: 1.04–2.09) after adjusted for variables confounders. Furthermore, after adjustment, in receiver operating characteristic (ROC) curve, the AUROC was 0.79 (95% CI = 0.75–0.82), 0.78(95% CI = 0.74–0.82) and 0.78(95% CI = 0.74–0.81) for presence of MetS according to NCEP-ATP III, international IDF and Iranian IDF, respectively. ROC analyses revealed that all of these three criteria for MetS are “moderately accurate” for the identification of increased stroke risk.

In conclusion, our results showed that MetS was associated with increased odds of stroke. Our findings implicate the importance of early identification, treatment, and ultimately prevention of the metabolic syndrome.

## Introduction

Stroke has been introduced as the most common cause of neurological and physical disability and also mortality in developing countries [1, 2]. Strokes can be classified into two main categories including ischemic and hemorrhagic strokes. According to the global statistics, ~ 71% of all strokes include ischemic stroke with impaired blood flow (infarction) to the brain or spinal cord [3]. The most important complications after a stroke consist of motor disability, sensory dysfunction, and dysphagia [4]. According to the annual estimation, there are 4.5 million deaths from stroke and more than 9 million stroke survivors worldwide [5].

Metabolic syndrome (MetS) could be defined as a significant risk factor for vascular diseases including stroke [6]. The major components of MetS are central obesity, elevated triglycerides, diabetes, increased blood pressure and reduced high-density lipoprotein) HDL) [7]. Age, sex, smoking, alcohol consumption and physical activity have been reported as associated factors with MetS [8]. Concerns about MetS as a public health problem is arising worldwide. The prevalence of MetS has been reported 14–30% globally [9]. Epidemiological studies suggest that MetS prevalence in Iran has increased to 30.8% in 2020 [10]. It is hypothesized that the inflammatory process in MetS plays a significant role in the proliferation of CVD, which can be due to the dysregulation of fat metabolism [9].

Of note, there are studies that have showed a positive and strong relationship between MetS and the risk of stroke. Two salient examples include: (1) A meta-analysis of thirteen cohort studies comprising 59,919 participants older than 60 years of age that showed MetS, especially low HDL-C and presence of more than one of the MetS components, were significantly associated with stroke recurrence (relative risk (RR) 1.46, 95% confidence interval (CI) 1.07–1.97) [11]; and (2) Another meta-analysis of 16 prospective cohort studies recruiting 116,496 cardiovascular diseases free participants, which demonstrated a significantly higher risk of incident stroke in those with MetS (RR:1.70, 95% CI: 1.49–1.95), and this increased risk was particularly more prominent among women and for ischemic rather than hemorrhagic stroke [12]. However, neither of these studies has been conducted on Iranian population particularly in large-scale cohort studies, so as to be able to reiterate such positive relationship in this special geographic area with prominent differences in ethnic, socio-economic, and especially dietary factors in comparison with other countries, which could potentially impact outcome measures of such epidemiological studies. Therefore, this study was aimed to assess the association between MetS and stroke in this specific region in the middle east to fulfill the knowledge gap regarding the differences in race and environmental factors, considering the high prevalence of metabolic syndrome and its effects on the progression of vascular diseases such as stroke.

## Methods

### Study population

Participants aged 35–70 years residing at Rafsanjan, a city in southeast Iran, have been selected to enroll in this cross-sectional study. The sample size was 9990 participants in Rafsanjan Cohort Study (RCS) [13], an arm of the Prospective Epidemiological Research Studies in Iran (PERSIAN) [14]. All of participants had completed questionnaires of demographical, medical, and habitual history. The protocol and questionnaires of this cross-sectional study were designed following the Persian cohort study protocols and under the supervision of the Iranian Ministry of Health and Medical Education (IMHME) [14]. In addition, they were approved by the Ethics Committee of Rafsanjan University of Medical Sciences with the Ethical code of IR.RUMS.REC.1400.111, and all methods were carried out following the relevant guidelines and regulations.

### Data collection

All of selected participants had filled out the questionnaires approved by Persian Cohort study under a standardized interview [14]. The questionnaires consisted of questions on demographical status (age, gender, education years and socio-economic status), opium and alcohol consumption, smoking, physical activity, blood pressure, body mass index (BMI), nutritional status, laboratory test, and history of disease. The history of stroke was identified through self-report. Blood pressure was measured twice for each participant from each arm. Anthropometric parameters including height, weight and waist circumference were measured according to a standard protocol [13].

Blood samples were collected after 12 h fasting from all participants. Fasting blood sugar (FBS), triglyceride (TG), and high-density lipoprotein cholesterol (HDL) levels were measured using Auto Analyzer (BT 1500, Biotechnica, Italy). The CBC assay was also performed using alpha cell counter (Nihon Kohden, Tokyo, Japan). All experiments were performed in Rafsanjan cohort laboratory.

Physical activity was measured by 22 questions about total hours reported for activities in 24 h and mentioned as Metabolic Equivalent of Task (MET) calculated for 24 h. It was categorized in three levels (low, moderate, and heavy, respectively, as ≤ 35.29, 35.30-40.32, and > 40.32 MET-hours per day) [14].

The nutritional status was measured for any subset of variables from the food frequency questioner (FFQ). The socio-economic status (Wealth score index: WSI) was estimated by multiple correspondence analysis (MCA) of the economic variables.

The metabolic syndrome prevalence was evaluated in participants according to three definitions including Adult Treatment Panel III (ATP-III), International Diabetes Federation (IDF) and Iranian criteria (NCEP-ATP III, international IDF, and IDF ethnic specific cut-off for Iranian population). Based on NCEP-ATP III, having three or more of the following criteria is considered as metabolic syndrome:

1- Waist circumference ≥ 102 cm in men and ≥ 88 cm in women,

2- Triglyceride level ≥ 150 mg/dl or receiving medication,

3- HDL level < 40 mg/dl in men and < 50 mg/dl in women or receiving medication,

4- Hypertension (systolic blood pressure ≥ 130 mmHg or diastolic blood pressure ≥ 85 mmHg) or receiving medication,

5- Hyperglycemia (fasting blood sugar ≥ 100 mg/dl or receiving medication).

Based on international IDF, waist circumference ≥ 94 cm in men and ≥ 80 cm in women plus at least two of the following criteria is considered as metabolic syndrome:

1- Triglyceride level ≥ 150 mg/dl.

2- HDL < 40 mg/dl in men and < 50 mg/dl in women or receiving medication.

3- Hypertension (systolic blood pressure ≥ 130 mmHg or diastolic blood pressure ≥ 85 mmHg) or receiving medication.

4- Hyperglycemia (fasting blood sugar ≥ 100 mg/dl or receiving medication).

Based on Iranian IDF, waist circumference ≥ 95 cm in both genders plus at least two of the following criteria is considered as metabolic syndrome:

1- Triglyceride level ≥ 150 mg/dl.

2- HDL < 40 mg/dl in men and < 50 mg/dl in women or receiving medication.

3- Hypertension (systolic blood pressure ≥ 130 mmHg or diastolic blood pressure ≥ 85 mmHg) or receiving medication,

4- Hyperglycemia (fasting blood sugar ≥ 100 mg/dl or receiving medication)[15].

### Statistical analyses

Relationship between MetS and stroke odds was assessed through the logistic regression models. Confounders were respectively included in models based on their assumptive strengths associated with MetS and stroke. Confounders including Age, gender, WSI, BMI, physical activity, smoking, alcohol consumption, opium use, nutrients status, family history of diabetes and blood pressure were measured in separate models at bivariate level. A p-value of < 0.05 was considered for multivariate analysis. Basic sociodemographic specifications (age, gender, education years, and WSI) were included in adjusted model 1. Afterwards, adjusted model 2 had additional adjustment for confounders including cigarette smoking and physical activity level. Adjusted model 3 was a combination of all variables in adjusted model 2 and had additional adjustment for family history of stroke, cardiac ischemic, MI, LDL, and nutrient’s status (total lipid, carbohydrate, energy, total fiber and protein). To clarify how stroke can be identified by MetS, receiver operating characteristic (ROC) curve analysis was applied. According to the method suggested by Swets et al. [16], the area under the ROC curve implies less accurate (0.5 < AUROC < 0.7), moderately accurate (0.7 < AUROC < 0.9), highly accurate (0.9 < AUROC < 1), and perfect (AUROC = 1) tests. All of the analyses were performed using State V.12. All p-values were two-sided, and p-values < 0.05 and 95% confidence intervals were considered as statistically significant.

## Results

Among 9991 participants in the baseline phase of the RCS, 9934 participants completed the medical questionnaire. Baseline characteristics of the participants are shown in Table 1.

**Table 1:** Demographic, selected medical, nutritional, and laboratory characteristics of study participants (n = 9934)

Characteristics	All (n = 9934)	Stroke (n = 153)	Non-Stroke (n = 9781)	P-Value
**Age- yr. no (%)**				< 0.001
35–45	3697(37.22)	17(11.11)	3680(37.63)	
46–55	3060(30.81)	32(20.92)	3028(30.96)	
≥ 56	3176(31.97)	104(67.97)	3072(31.41)	
Mean ± SD	49.94 ± 9.56	57.71 ± 8.07	49.82 ± 9.54	< 0.001
**Gender- no (%)**				0.717
Female	5310 (53.45)	84 (54.90)	5226 (53.43)	
Male	4624 (46.55)	69 (45.10)	4555 (46.57)	
**WSI**				0.003
Median (IQR)	-0.034 (-0.61-0.58)	-0.145 (-0.78-0.48)	0.034 (-0.61-0.58)	
**Education years – n (%)**				< 0.001
≤ 5	3484 (35.09)	93 (61.18)	3391 (34.68)	
6–12	4820 (48.54)	45 (29.61)	4775 (48.84)	
≥ 13	1625 (16.37)	14 (9.21)	1611 (16.48)	
Mean ± SD	8.52 ± 5.05	5.42 ± 4.88	8.57 ± 5.04	< 0.001
**Physical activity- no (%)**				0.018
Low	3850 (38.76)	75 (49.02)	3775 (38.60)	
Moderate	5053 (50.87)	61 (39.87)	4992 (51.04)	
Heavy	1031 (10.38)	17 (11.11)	1014 (10.37)	
Mean ± SD	38.79 ± 6.32	37.86 ± 7.42	38.81 ± 6.30	0.070
**BMI- no (%)**				0.047
< 25	2868 (28.89)	39 (25.49)	2829 (28.94)	
25-29.9	4070 (41.00)	54 (35.29)	4016 (41.08)	
≥ 30	2990 (30.12)	60 (39.22)	2930 (29.97)	
Mean ± SD	27.81 ± 4.92	28.46 ± 5.95	27.81 ± 4.90	0.103
**Alcohol consumption- no (%)**				0.759
Yes	993 (10.02)	14 (9.27)	979 (10.03)	
No	8921 (89.98)	137 (90.73)	8784 (89.97)	
**Cigarette smoking-no (%)**				0.036
Current	1679 (16.94)	25 (16.56)	1654 (16.94)	
Former	864 (8.71)	22 (14.57)	842 (80.62)	
Never	7371 (74.35)	104 (68.87)	7267 (74.43)	
**Opium consumption- no (%)**				0.110
Yes	2345 (23.65)	44 (29.14)	2301 (23.57)	
No	7569 (76.35)	107 (70.86)	7462 (76.43)	
**Cholesterol**				< 0.001
Mean ± SD	198.66 ± 38.04	186.87 ± 41.70	198.85 ± 37.96	
**Triglycerides**				0.99
Mean ± SD	169.16 ± 110.74	169.03 ± 99.67	169.16 ± 110.91	
**LDL cholesterol**				< 0.001
Mean ± SD	108.18 ± 30.29	99.28 ± 32.47	108.32 ± 30.23	
**HDL cholesterol**				< 0.001
Mean ± SD	57.75 ± 10.87	54.86 ± 11.87	57.80 ± 10.85	
**FBS**				0.003
Mean ± SD	113.30 ± 39.14	122.65 ± 44.96	113.15 ± 39.03	
**Waist**				< 0.001
Mean ± SD	95.91 ± 11.50	100.01 ± 12.79	95.84 ± 11.47	
**Systolic blood pressure**				< 0.001
Mean ± SD	106.29 ± 17.04	112.49 ± 17.15	106.19 ± 17.02	
**Diastolic blood pressure**				0.106
Mean ± SD	70.64 ± 10.24	71.97 ± 10.20	70.62 ± 10.24	
**Diabetes- no (%)**				< 0.001
Yes	1933 (19.46)	60 (39.22)	1873 (19.15)	
No	8001 (80.54)	93 (60.87)	7908 (80.85)	
**MI- no (%)**				< 0.001
Yes	296 (2.98)	19 (12.42)	277 (2.83)	
No	9638 (97.02)	134 (87.58)	9504 (97.17)	
**Cardiac ischemic disease- no (%)**				< 0.001
Yes	870 (8.76)	43 (28.10)	828 (8.46)	
No	9064 (91.24)	110 (71.90)	8954 (91.54)	
**Family history of stroke- no (%)**				0.005
Yes	1753 (17.65)	40 (26.14)	1713 (17.51)	
No	8181 (82.35)	113 (73.86)	8068 (82.49)	
**Nutrient’s status**				
**Total lipid (gr/day)**				< 0.001
Mean ± SD	55.63 ± 23.56	48.80 ± 21.97	55.74 ± 23.57	
**Carbohydrate (gr/day)**				0.005
Mean ± SD	324.98 ± 120.64	297.98 ± 139.36	325.41 ± 120.29	
**Energy (kcal/day)**				0.001
Mean ± SD	2042.68 ± 728.84	1851.24 ± 821.92	2045.69 ± 726.93	
**Total Fiber (gr/day)**				0.39
Mean ± SD	21.84 ± 9.69	21.17 ± 12.24	21.85 ± 9.64	
**Protein (gr/day)**				0.008
Mean ± SD	68.74 ± 26.49	63.14 ± 30.45	68.83 ± 26.42	

Abbreviations: Wealth score index (WSI); body mass index (BMI); Fasting blood sugar (FBS), high density lipoprotein cholesterol (HDL), and Myocardial Infarction (MI).

The prevalence of stroke among all participants was 1.54%. The average age of people who had a stroke was significantly higher than people without stroke (P < 0.001), and the highest percentage of stroke was seen in people over 56 years old. People who had low physical activity had a significantly higher percentage of stroke (49.02%) compared to others (P = 0.018).

The prevalence of stroke was significantly higher in participants with less than 6 years of education, obesity (BMI = ≥ 30), sedentary lifestyle, and people with history of diabetes, MI, cardiac ischemic disease, and family history of stroke. Also, the median of WSI in participants with stroke was lower than non-stroke population (Table 1).

The mean of waist, fasting glucose, and systolic blood pressure was significantly higher and HDL cholesterol was significantly lower in people with history of stroke compared with people without history of stroke. Also, intake of nutrients including lipid, carbohydrate, energy, and protein were remarkably less in people with history of stroke compared with others (Table 1).

Prevalence of metabolic syndrome in the RCS participants was 38.31, 34.37 and 31.57 according to international IDF, NCEP-ATP III and Iranian IDF, respectively. According to criteria, the prevalence of stroke was higher in people with MetS disorder compared to people without MetS disorder at baseline. History of stroke in people with MetS disorder was 60.78, 59.87 and 50.98 according to the international IDF, NCEP-ATP III and Iranian IDF, respectively (Table 2).

**Table 2:** Associations between the metabolic syndrome classification and stroke in study participants (n = 9934)

Metabolic syndrome Indicators	All N (%)	Stroke N (%)	Non-Stroke N (%)	p-value
**Metabolic syndrome indices (ATP-III)**				< 0.001
yes	3397 (34.37)	91 (59.87)	3306 (33.97)	
no	6488 (65.63)	61 (40.13)	6427(66.03)	
**Metabolic syndrome indices (international IDF)**				< 0.001
yes	3798 (38.31)	93 (60.78)	3705 (37.96)	
no	6116 (61.69)	60 (39.22)	6056 (62.04)	
**Metabolic syndrome indices (Iranian IDF)**				< 0.001
yes	3130 (31.57)	78 (50.98)	3052 (31.27)	
no	6784 (68.43)	75 (49.02)	6709 (68.73)	

### Metabolic syndrome (NCEP) and stroke odds

Table 3 presents the association of MetS with stroke, using the crude and three adjusted models. In the crude regression model, the odds of stroke according to NCEP-ATP III (odds ratio (OR): 2.90, 95%CI 2.09 to 4.02) and international IDF (odds ratio (OR): 2.53, 95%CI 1.85 to 3.51) are almost triple greater among people with MetS compared with other people. This association decreased to about twice after adjustment for confounding variables including age, gender, education years, and wealth status index (adjusted model 1). The corresponding adjusted ORs calculated for people with MetS compared with people without MetS were 2.13 (95% CI 1.48 to 3.06) and 1.82 (95% CI 1.28 to 2.60), respectively, for NCEP-ATP III and international IDF (Table 3).

**Table 3:** Odds ratios (95% confidence interval) for Stroke according to metabolic syndrome

metabolic syndrome indices	Crude model	Adjusted model 1	Adjusted model 2	Adjusted model 3
	OR (95%CI)^a^	OR (95%CI) ^b^	OR (95%CI)^c^	OR (95%CI) ^d^
**Metabolic syndrome on the basis of ATP-III definition**				
No	1	1	1	1
Yes	2.90 (2.09–4.02)	2.13 (1.48–3.06)	2.18 (1.51–3.15)	1.89 (1.30–2.74)
**Metabolic syndrome on the basis of international IDF definition**				
No	1	1	1	1
Yes	2.53 (1.85–3.51)	1.82 (1.28–2.60)	1.87 (1.30–2.67)	1.66 (1.15–2.40)
**Metabolic syndrome on the basis of Iranian IDF definition**				
No	1	1	1	1
Yes	2.29 (1.66–3.15)	1.63 (1.17–2.29)	1.66 (1.18–2.33)	1.48 (1.04–2.09)

^a^ The baseline model is not adjusted.

^b^ The adjusted model 1 is adjusted for confounding variables including age (continuous variable), gender (male/ female), education years (continuous variable), and wealth status index (continuous variable).

^C^ The adjusted model 2 has additional adjustment for confounders including cigarette smoking (yes/no) and physical activity level (continuous variable).

^d^ The adjusted model 3 has additional adjustment for family history of stroke (yes/no), cardiac ischemic disease, MI (yes/no), LDL (continues variable) and nutrient’s status (total lipid, carbohydrate, energy, total fiber, and protein as continues variables).

Adjusted model 2 included all variables considered in adjusted model 1, plus cigarette smoking and physical activity level. However, after adjusting for the variables (adjusted model 2), the obtained results showed no appreciable change and the mentioned association of stroke and MetS according to NCEP-ATP III (odds ratio: 2.18, 95%CI 1.51 to 3.15) and international IDF (odds ratio (OR):1.87, 95%CI 1.30 to 2.67) was observed again. Finally, adjusted model 3 included all variables considered in adjusted model 2, plus family history of stroke as potential confounder and also history of cardiac ischemic disease, history of MI, LDL, and nutrient’s status (total lipid, carbohydrate, energy, total fiber and protein), which could act as potential confounder in the causal pathways describing the association of MetS with stroke. However, after adjusting for the variables (adjusted model 3), this association remained stable, although it decreased slightly. In addition, the odds of stroke according to Iranian IDF in all models were higher among people with MetS compared with people without MetS. These odds in all models were lower than definitions of metabolic syndrome according to NCEP-ATP III and international IDF (Table 3).

**Fig. 1:**
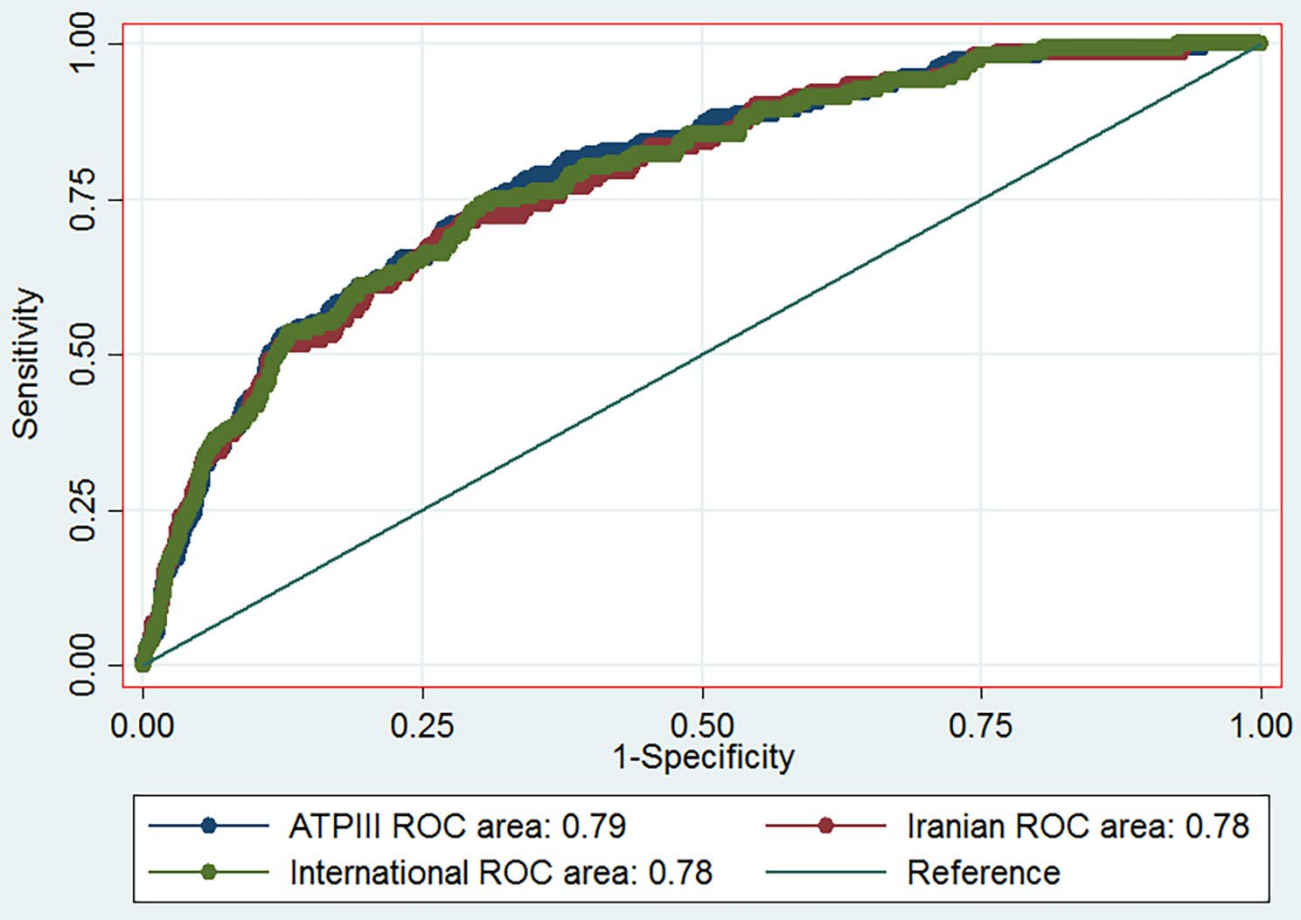
ROC curve of effective metabolic syndrome according to difference criteria for the identified stroke

Furthermore, after adjustment, receiver operating characteristic (ROC) curve analysis was employed to clarify how stroke can be identified by definition of metabolic syndrome according to different criteria. In the ROC analysis predicting the presence of stroke, the AUROC was 0.79 (95% CI = 0.75–0.82), 0.78 (95% CI = 0.74–0.82) and 0.78 (95% CI = 0.74–0.81) for presence of metabolic syndrome according to NCEP-ATP III, international IDF and Iranian IDF, respectively (Fig. 1; Table 4); there was no statistically significant difference among these three criteria in this regard (p = 0.148) (Table 3). ROC analyses revealed that MetS according to NCEP-ATP III, international IDF and Iranian IDF is “moderately accurate” for the identification of stroke.

**Table 4:** Effective metabolic syndrome according to different criteria for the screening of stroke using ROC analysis

metabolic syndrome	AUROC	Standard Error	95% CI	p-Value
NCEP-ATP III	0.79	0.018	(0.75–0.82)	0.148
International IDF	0.78	0.019	(0.74–0.82)	
Iranian IDF	0.78	0.019	(0.74–0.81)	

Finally, ROC analyses revealed that metabolic syndrome according to different criteria are moderately accurate for the effective identification of stroke.

## Discussion

According to the results of this study, history of stroke in people with MetS disorders was more in comparison with people without MetS at baseline according to all three criteria. Accordingly, the presence of MetS has been associated with an increased risk of prevalent stroke in the existing literature. For example, in the National Health and Nutrition Examination Survey among 10,357 subjects, the prevalence of MetS was significantly higher in people with a self-reported history of stroke (43.5%) than in people with no history of vascular disease (22.8%). The results of the mentioned study, indicate a strong, consistent relationship of the metabolic syndrome with prevalent MI and stroke [17]. Another previous study has shown that metabolic syndrome is associated with self-reported history of stroke and myocardial infarction and stroke together [17].

In the present study, after adjusting all variables, which could act as potential confounders in the causal pathways describing the association of MetS with stroke, the odds of stroke according to three definitions in all models were higher among people with MetS compared with non- MetS people. Similarly, the results of the study by Mottillo et al. [18] has shown that metabolic syndrome was associated with a 2-fold increase in risk of stroke. In a case-control study, Milionis et al. [19] demonstrated that patients with MetS had higher odds for a first-ever ischemic non-embolic stroke. In a study conducted by Chen et al. [20] in line with this study, MetS was associated with higher incidence of stroke. So, it can be stated that the metabolic syndrome is a risk factor for stroke that seemingly has an underlying metabolic causation. Central obesity is the centerpiece of the metabolic alterations. Accordingly, increased abdominal adiposity contributes to dyslipidemia, hyperglycemia, and hypertension [21].

Further and more importantly, though, is the fact that we found significant differences the stroke and the non-stroke groups based on some demographic variables. The prevalence of stroke was significantly more in elderly, obese, smoker, and sedentary people who had history of diabetes, MI, cardiac ischemic and family history of stroke. In line with these results, some studies in Iran and other countries, reported similar results [22–28]. Of utmost importance, two high-quality meta-analysis studies including one study reviewing 16 prospective cohort studies with a total number of 116,496 participants who were initially free of cardiovascular diseases [12] and the second one comprising 13 cohort studies recruiting an overall 59,919 elderly people [11], concluded that in comparison with the non-Mets group, people with MetS had a significantly higher risk of incident stroke (RR = 1.70) and recurrent stroke (RR = 1.46), respectively. These findings were in accordance with our results even after all implemented adjustments and in a population with different geographical and demographical characteristics, providing a reasonable credit for our findings and conclusions based on our audit objectives. Notwithstanding, neither did we discriminate among gender differences and various stroke subtypes like subgroup analyses in the former study showing a significantly higher risk of ischemic stroke (RR = 2.12, 95% CI: 1.46–3.08) than hemorrhagic stroke (RR = 1.48, 95% CI: 0.98–2.24) and more sensitivity of women (with an RR of 1.83, 95% CI: 1.31–2.56) than men (RR = 1.47 (95% CI: 1.22–1.78) to the increased stroke risk, nor had we examined the effect size based on the MetS components like the latter study reporting an increased stroke recurrence more prominent in the low HDL-C level group (RR 1.32, 95% CI 1.11–1.57, *p* = 0.002) and in the presence of ≥ 2 metabolic syndrome components (RR 1.68, 95% CI 1.44–1.94, *p* < 0.001).

Finally, in our study, the mean of waist circumference (WC), FBS and systolic blood pressure were significantly higher among people with history of stroke and HDL cholesterol level was lower in this group compared with people without history of stroke. In line with our study, in a Nationwide Population-Based Study with 21,749,261 participants conducted by Cho et al. WC demonstrated a significant linear relationship and was powerful enough to predict the risk of ischemic stroke [29], and a systematic review and dose–response meta-analysis of 4.43 million participants reported that overweight and obesity increase the stroke risk in a J-shaped dose–response manner [30]. Similarly, a meta-analysis study by Sarikaia et al. [31] reported that reductions of elevated systolic blood pressure (SBP) and diastolic blood pressure (DBP) are associated, respectively, with stroke reduction by 25% and 50%. Also, the results of a study conducted by Muñoz-Rivas et al. [32] showed that in people with diabetes mellitus type 2, the risk of ischemic stroke increases more than twice after adjusting for other risk factors, which is consistent with the results of our study. Such being the case, fasting blood glucose, blood pressure and body weight that are considered to be related to components of MetS should be well controlled to reduce the risk of recurrent stroke in patients with history of stroke and also to prevent stroke in individuals without history of stroke.

This study has strengths and limitations. Population-based nature study, large sample size, extensive data collection for the exposure of interest (MetS), potential confounders, and its standardized methodology are the main strengths of this study. However, the study has some limitations too. The main limitation of our study was its cross-sectional design that refrained us to derive any causal inferences. Furthermore, our data source was self-report, and the ischemic and hemorrhagic types of strokes were not separated.

## Conclusion

In conclusion, our results showed that metabolic syndrome was associated with the increased odds of stroke. Our findings implicate the importance of early identification, treatment, and ultimately prevention of the metabolic syndrome.

## Data Availability

The datasets used during the current study are available on the Persian Adult Cohort Study Center, Rafsanjan University of Medical Sciences, Iran. The data is not available publicly. However, upon a reasonable request, the data can be obtained from the corresponding author.
